# Rare Taxa as Key Drivers of Soil Multi-Nutrient Cycling Under Different Crop Types

**DOI:** 10.3390/microorganisms13030513

**Published:** 2025-02-26

**Authors:** Qingmiao Yang, Hanwen Liu, Biao Tang, Chunxiao Yu, Shide Dong, Yang Li, Guangxu Cui, Yi Zhang, Guangmei Wang

**Affiliations:** 1 School of Life Sciences, Ludong University, Yantai 264025, China; yangqingmiao79@163.com; 2 CAS Key Laboratory of Coastal Environmental Processes and Ecological Remediation, Yantai Institute of Coastal Zone Research, Chinese Academy of Sciences, Yantai 264003, China; hwliu@yic.ac.cn (H.L.); cxyu@yic.ac.cn (C.Y.); sddong@yic.ac.cn (S.D.); yangli@yic.ac.cn (Y.L.); gxcui@yic.ac.cn (G.C.); yizhang@yic.ac.cn (Y.Z.); 3 CCCC-FHEC Ecological Engineering Co., Ltd., Shenzhen 518106, China; tb1595523@163.com

**Keywords:** rare microbial taxa, abundant microbial taxa, soil multi-nutrient cycling index, crop types

## Abstract

Soil microorganisms are crucial for nutrient cycling, with abundant and rare taxa playing distinct roles. However, the mechanisms by which soil microbes influence nutrient cycling under different crop types remain unclear. In this study, we investigated the network structure, diversity, and microbial composition of croplands in the Yellow River Delta, focusing on four primary crops: soybean, maize, cotton, and sorghum. The findings revealed that the co-occurring network structure of sorghum planting-soils exhibited greater complexity than other crop types. Bacterial alpha diversity in cotton-planting soil is the highest and susceptible to environmental variations. The diversity of both rare and abundant taxa responds differently to soil nutrients depending on the crop type. While abundant taxa play a crucial role in soil multi-nutrient cycling, rare taxa are key drivers of variations in nutrient cycling expression. The diversity of rare taxa showed a strong correlation with critical nutrients. Structural equation modeling revealed that the alpha diversity of rare bacterial and fungal taxa significantly influenced the soil multi-nutrient cycling index (*MNC*). Specifically, higher Shannon indices of rare bacterial taxa were associated with lower *MNC*, while the opposite was true for soil fungi. Soil organic carbon and soil total nitrogen are the key factors influencing alpha diversity in rare bacterial and fungal taxa. Moreover, this study provides new insights into the role of rare soil microbial diversity in the nutrient cycling of agricultural ecosystems.

## 1. Introduction

Soil salinization is a significant challenge faced by modern agriculture, and enhancing the agricultural utilization of saline-alkali land can contribute to addressing food security issues in the context of population growth and climate change [1]. According to the latest data from the FAO, around 1 billion hectares of land, representing 7% of the Earth’s total land area, are currently affected by salinity [2]. Salt-affected soils are estimated to comprise over 10% of the world’s arable land [3]. The urgent need for sustainable farming practices is evident in the proper management of saline soils, preservation of soil health, and fulfillment of global food requirements.

The microbiome serves as a crucial barometer of ecosystem health and exhibits significant sensitivity to alterations in the environment [4]; they are essential for geochemical cycles and various aspects of biological health [5,6]. The microbial community can be divided into two groups, rare and abundant taxa, based on their abundance. Referring to previous studies [4], we classified the soil bacteria and fungi OTUs into two taxa: abundant (>0.05%) and rare (<0.001%). In agroecosystem, abundant taxa are vital in maintaining and stabilizing soil microbial communities [7]. Meanwhile, the presence of rare taxa enhances the resilience and resistance of these microorganisms to environmental disturbances [8]. In response to environmental stressors like soil acidification, salinity, and continuous cropping, rare taxa can bolster the functional redundancy of microbial communities [8,9,10]. Microbial diversity, encompassing rare and abundant taxa in the root zones of various crops, is pivotal for crop health and development. It has been demonstrated that rare taxa have narrower ecological niches, are more susceptible to environmental influences, and can respond more clearly to changes in soil status than abundant taxa [11]. However, the responses of both abundant and rare taxa to environmental changes are not always consistent. Different root structures and different root secretions of crops grown can lead to differences in soil structure and adjustments in the soil microbial community. Different crop types also mean differences in land-use intensity and field management practices [12]. The unique requirements and resource utilization patterns of different crops can influence the composition and diversity of these microbial communities. Therefore, understanding the diversity of these microbial taxa is essential for improving arable land- and resource-use efficiency in agricultural soils [13]. There are still gaps regarding the role of specific crop types, especially rare and abundant taxa, in soil microbial diversity [10,12]. There is a need for specific exploration of microbial diversity, species composition, and co-occurrence network structure among major crop types in a specific area.

An effective way to assess soil health is by measuring various nutrient cycling processes, known as soil multi-nutrient cycling [14]. The physical and chemical properties of soil are closely connected to microorganisms, which play a crucial role in soil nutrient cycling [15,16]. Biological information, rather than physical or chemical characteristics, can indicate soil health because microbes respond more rapidly to environmental changes. Numerous studies have proven that rare taxa are critical factors in the adjustment of ecosystem functions, especially with key functional genes related to soil nutrient cycling. In arid agricultural regions, rare taxa play crucial roles in soil processes like carbon, nitrogen, cycling, and soil carbon sequestration [17,18]. For example, some rare taxa may develop into dominant species under the influence of biotic or abiotic factors, providing unique ecological functions [11]. However, studies on the differential responses of rare and abundant taxa, induced by planting crop variables in agroecosystems, are largely unknown. Chemicals (e.g., organic acids, sugars, etc.) released by the root systems of different crops affect the growth and distribution of microorganisms [19], and rare taxa may be uniquely adapted to participate efficiently in nutrient cycling within the inter-root microbial community. Meanwhile, under agroecosystems, studies have been conducted to demonstrate the effects of soil microorganisms on multiple nutrient cycling indices, but fewer studies have been conducted on rare and dominant communities within soil microorganisms. Therefore, it is imperative to elucidate how these rare taxa function under different agricultural management practices, particularly their involvement in the multi-nutrient cycling of soil. Further research is required across various agroecosystems to comprehend these dynamics, particularly in soils that pose challenges such as salinity.

The Yellow River Delta (YRD) is a prime example of saline soil amelioration [20]. The saline-alkali landscape in this area exhibits significant salinization variability due to geographic location, groundwater levels, and human activities [21,22]. The widespread presence of saline-alkali arable land in Dongying characterizes it as a quintessential part of the YRD, where soil salinization poses a significant challenge to agricultural development. Previous research conducted on soil health of the YRD predominantly focused on microbial populations, heavy metals, organic pollutants, nutrient imbalances, and salinization [23,24]. Research on soil microorganisms, particularly their mechanisms for influencing nutrient cycling about different crop types, is limited in the YRD. Varied soil types, numerous crops, a complex cropping system, and differences in the health of agricultural land characterize the region. There is a lack of understanding regarding how rare and abundant soil taxa regulate nutrient cycling. This study aimed to improve our understanding of a typical agricultural field in the YRD region, especially concerning light salinization. The analysis encompasses soil physical and chemical properties, microbial diversity, community composition, multi-nutrient cycling index, and the intricate interrelationships among these factors. The objective is to discern the disparities between the environment and microbiome under varying crop types. Furthermore, this research aimed to elucidate the mechanism by which microorganisms mediate changes in multi-nutrient cycling across different crop types. We hypothesized: (1) Soil microbial diversity and community composition vary among different crop types; (2) crop types impact the diversity response of both rare and abundant taxa to environmental factors; (3) rare taxa significantly influence *MNC*.

## 2. Materials and Methods

### 2.1. Study Area and Data Collection

**Figure 1:**
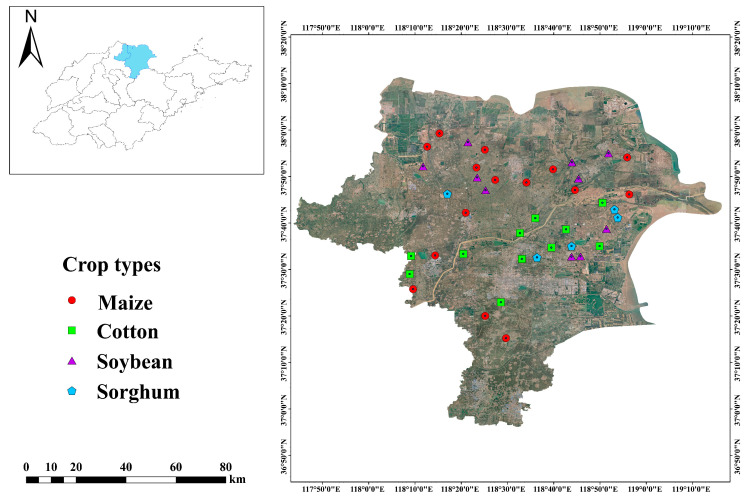
Location of sampling sites (*n* = 41) in the vicinity of the Yellow River Delta region.

The YRD, located at coordinates 37°25 E–37°99 E, 118°15 N–118°94 N, is a contemporary depositional plain formed through significant sediment accumulation. It is distinguished by its surface-level groundwater, inferior soil texture, and a high evaporation rate relative to precipitation. This makes it one of the most critical coastal saline-alkali regions in China’s mild temperate zone [22]. The study was conducted long-term on low-saline farmland, which was primarily planted with cotton, soybeans, sorghum, and maize. Due to the diverse distribution of soil types, organic matter, and salinity in this area, we employed a stratified random sampling method to select appropriate geographical scales for the random placement of sample plots and the recording of soil data. And soil sampling was conducted in September of 2022 (Figure 1). Four crop types were planted in 41 sample plots: soybean (*n* = 10), cotton (*n* = 11), sorghum (*n* = 5), and maize (*n* = 15). Five soil cores were randomly selected from each sample plot. Ice packs and an insulated container were utilized, and samples were extracted from five randomly selected cores at 0 to 20 cm depth in each sampling plot. These samples were then combined to create a representative soil sample for laboratory analysis.

We measured electrical conductivity (EC), pH, soil bulk density (BD), total nutrients, available nutrients, and soil organic carbon content, with the methods provided in the Supplementary Material.

### 2.2. Amplicon Sequencing

Microbial DNA extraction was performed using the Fast DNA SPIN Kit for Soil from 0.50 g of fresh soil (MP Biomedicals LLC, Solon, OH, USA), following the procedure outlined in the soil microbial DNA extraction kit. Before PCR amplification, the concentration and purity of the DNA were examined by measuring the absorbance ratios of microbial DNA at 260/230 nm and 260/280 nm with a NanoDrop ND-2000 spectrophotometer (NanoDrop Technologies Inc., Wilmington, DE, USA). Shanghai, China’s Majorbio Bio-Pharm Technology Co., Ltd. performed the subsequent sequencing. The ABI GeneAmp^®^ 9700 PCR amplifier was used in the TransGen AP221-02: TransStart Fastpfu DNA polymerase reaction system to perform the polymerase chain reaction (PCR) amplification of bacterial and fungal DNA. Primers 515F and 907R [25] were used for bacterial amplification, while the identical primer pairs were used for fungus amplification. Fungal DNA was amplified in 2008 using the primer pairs ITS86F and ITS4R [26]. To determine the original gene sequences, each sample was put through the PCR using primers that carried a barcode for a particular sequence.

Specific information on high-throughput sequencing and bioinformatics analyses were added in the Supplementary Material.

### 2.3. Soil Multi-Nutrient Cycling Index

Soil multi-nutrient cycling represents the most critical process regulating and supporting the functioning of terrestrial ecosystems [27]. To evaluate this process, the *MNC* index was computed. After min-max normalization, the MNC was calculated as the mean of seven measured nutrient properties which were soil organic carbon (SOC), total nitrogen (TN), total phosphorus (TP), total potassium (TK), Olsen-phosphorus (Olsen-P), available potassium (Avail-K), and nitrate nitrogen (NO_3_^−^-N). This method is frequently employed to quantify multi-functionality at the ecosystem level and has recently been utilized to elucidate the correlation between microbial diversity and soil nutrient cycling [4]. The equation used to calculate *MNC* was as follows:

$$N_{or}=\frac{X-X_{min}}{X_{max}-X_{min}}$$

$$MNC=\frac{\sum_{i=1}^{n}N_{ori}}{n}$$

The variable “*N_or_*” is the normalized variable, the variable “*X*” is the measured value of the target variable, the variable “*X_min_*” is the minimum value observed in all samples, and the variable “*X_max_*” is the maximum value observed in all samples. The variable “*n*” is the number of variables. “*N_ori_*” is the normalized value of each individual sample.

### 2.4. Data Analyses

ArcGIS was used to plot sample sites for the four crop types. The one-way ANOVA using Duncan’s test was used to examine the variations in soil physicochemical characteristics among the four crop types. The “vegan” package (2.6-8) evaluated the Bray–Curtis distance nonmetric multidimensional scaling (NMDS) analyses and alpha diversity indices. The “ggplot2” package (3.5.1) was utilized to show the results. The selection of the Pielou, Richness, and Shannon indices reflects comprehensive information on community diversity, evenness, and species abundance. It provides an understanding of the characteristics of species distribution and community stability under different environmental conditions. To determine whether alpha diversity showed distinct crop types of soil, the Kruskal–Wallis test was utilized. The Bray–Curtis NMDS analysis helps to discover potential community structures between samples and presents the relationships between samples in a graphical form. The Adonis function was used in a permutational multivariate analysis of variance (PERMANOVA) using 999 permutations to examine the influence of environmental factors on differences in community structure, and to assess significant differences across several crop types of soil. A co-occurrence network based on the Spearman correlation matrix was built using the “picante” package (1.8.2) to clarify microbial co-linearity patterns. The top 80% of operational taxonomic units (OTUs) for fungi and bacteria, respectively, were kept before the network was built. The network was then visualized using the Gephi software (https://gephi.org/ (v.0.9.2)), which enables the display of such networks, and correlations with an absolute r value larger than 0.60 and a *p*-value less than 0.05 were kept for the Spearman correlation matrix. A mantal test analyzed the relationship between soil microbial diversity and environmental attributes. The “multifunc” package (0.9.4) was used to calculate the topology of the co-occurrence network. Robustness and cohesion indices of the co-occurrence network were computed using the “igraph” package (2.1.1). We used Pearson’s correlation analysis to evaluate how rare and abundant taxa diversity behaved differently to soil nutrients under different crop types by the “cor. test” function in “stats.” [28]. A structural equation modeling (SEM) script was established using AMOS (v.26) in the SPSS software (v.27) to further evaluate the causal relationship between soil salinity, the rare taxa alpha diversity of soil microorganisms, and MNC. The Chi-square/degrees of freedom (x^2^/df) and root-mean-square error of approximation (RMSEA) were used to estimate the SEM fitness. The Shannon index of rare and abundant taxa was used to evaluate the alpha diversity of the community. Finally, the model was visualized in BioRender (https://app.biorender.com/ accessed on 30 May 2024) and AI (2020) program. Finally, we used random forest models to analyze the critical factors in the process of multi-nutrient cycling as influenced by the rare taxa diversity of soil bacteria and fungi.

## 3. Results

### 3.1. Soil Physicochemical Properties Under Different Crop Types

The physicochemical characteristics of the soil differed significantly among crop types (Table 1). In particular, significant differences were observed in soil salinity and soil water content among soybean (0.76‰, 11.51%), sorghum (1.34‰, 18.70%), cotton (1.69‰, 16.29%), and maize (0.74‰, 14.07%). Notably, soil salinity in cotton and sorghum was significantly higher than the other two crop types (*p* < 0.05). Compared with sorghum, the planting soils of cotton, maize, and soybeans showed lower SOC, TN, available nitrogen (NH_4_^+^-N and NO_3_^−^-N), and TK (*p* < 0.05); additionally, soybean soils had significantly lower NH_4_^+^-N, TK, and TN (*p* < 0.05). The differences in soil nutrient availability (AN: TN, Avail-K: TK, and Olsen-P: TP) were examined among four crops, and results showed that cotton-planting soils (8.41, 1.19, 1.01) had the highest nutrient accessibility (*p* < 0.05). Furthermore, cotton-planting soils (1.19) had a significantly higher Avail-K: TK ratio compared to soybean (0.69) and sorghum (0.62) (*p* < 0.05). The soils used for cotton-planting had the greatest nutrient availability, particularly in potassium. Importantly, sorghum-planting soils (0.48) had the highest soil multi-nutrient cycling index (MNC).

### 3.2. Diversity and Composition of Soil Microbial Communities

**Figure 2:**
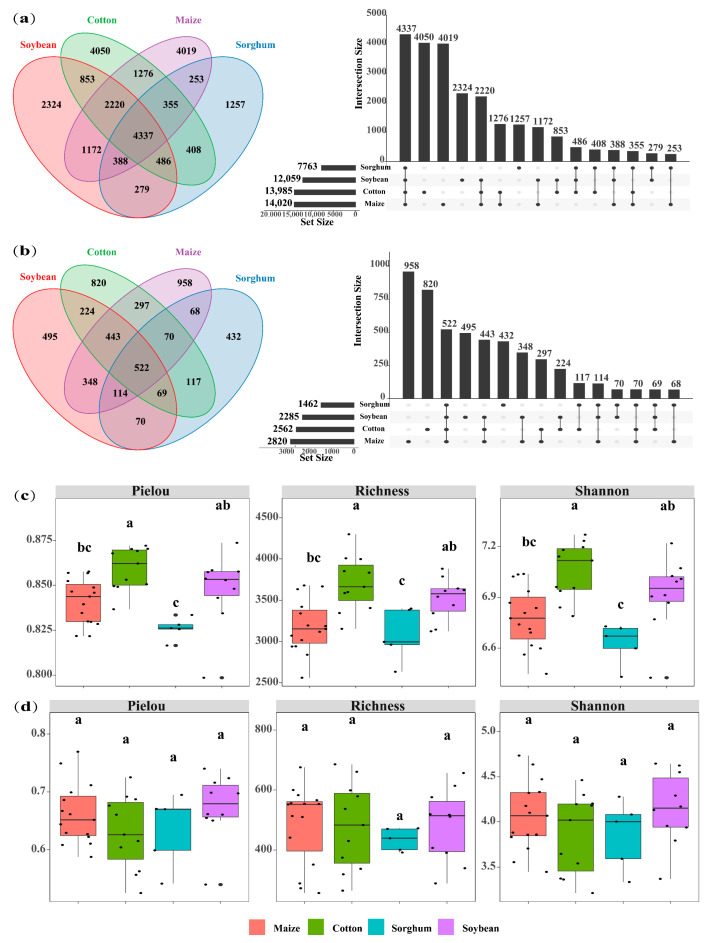
Venn plot shows the overlap of bacteria (**a**) and fungi (**b**) in four crops (cotton, maize, soybean, and sorghum). UpSet plot of the size of the intersection for each crop, indicating the number of microbial species shared between the different crop types. Boxplots show the alpha diversity indices (Pielou index, richness index, and Shannon index) for bacteria (**c**) and fungi (**d**) in the four crop types (maize, cotton, sorghum, and soybean). The letters above the boxes indicate significant differences between the crop types (*p* < 0.05).

**Figure 3:**
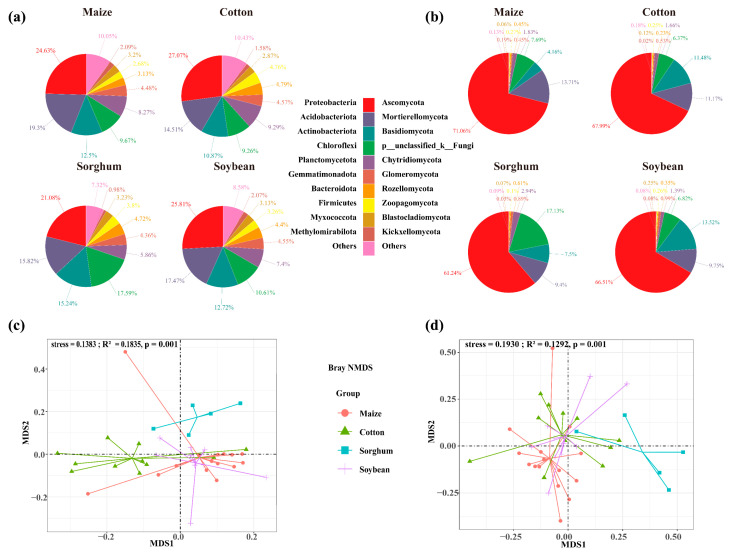
Relative abundances of soil bacterial (**a**) and fungal (**b**) communities at phylum level in each group. Bray–Curtis distance nonmetric multidimensional scaling (NMDS) analyses of the bacteria (**c**) and fungi (**d**) clustered by crop types.

The soils used for planting soybean, sorghum, cotton, and maize had 7763, 12,059, 13,985, and 14,020 OTUs of soil bacteria, respectively. The number of OTU in endemic soil bacterial species was higher in maize (4019 OTUs) and cotton (4050 OTUs) (Figure 2a). Crop types significantly impact the diversity of soil bacterial communities, with cotton-planting soils exhibiting greater bacterial diversity than those used for other crops (*p* < 0.05). Specifically, cotton-planting soils demonstrated significantly higher bacterial Pielou (0.86), richness (3704), and Shannon (7.06) index compared to maize, sorghum, and soybean-planting soils (*p* < 0.05). Across all crop types, the alpha indices consistently followed the trend: cotton > soybean > maize > sorghum (Figure 2c). The top ten bacterial phyla identified in the soil were *Proteobacteria*, *Acidobacteriota*, *Actinobacteria*, *Chloroflexi*, *Planctomycetes*, *Gemmatimonadota*, *Bacteroidota*, *Firmicutes*, *Myxococcota*, and *Methylomirabilota*. In soils of soybeans, sorghum, maize, and cotton, the overall abundance of dominant phyla was 91.4%, 92.7%, 89.6%, and 89.9%, respectively. Sorghum-planting soils had the highest relative abundance of dominating bacterial phyla. Cotton, maize, and sorghum-planting soil had the most significant relative abundances of *Proteobacteria* (27.07%), *Acidobacteriota* (19.30%), and *Actinobacteria* (15.24%), respectively (Figure 3a).

Soil fungal OTUs in soybean, cotton, maize, and sorghum-planting soils were 2285, 2562, 2820, and 3508, respectively. Similarly, the number of soil fungal endemics of cotton-planting (820 OTUs) and maize-planting (958 OTUs) exhibited the highest (Figure 2b), suggesting that these crops may influence the soil environments to promote the growth of soil microbial endemic species. Nevertheless, there was no significant variation in soil fungus alpha diversity indices among crop types (*p* > 0.05) (Figure 2d). *Ascomycota*, *Mortierellomycota*, *Basidiomycota*, *p_unclassified_k_Fungi*, *Chytridiomycota*, *Glomeromycota*, *Rozellomycota*, *Zoopagomycota*, *Blastocladiomycota*, and *Kickxellomycota* were identified as the ten dominant phyla of soil fungi. Among the four crop types, *Ascomycota* showed the highest significant relative abundance of dominating phyla. In contrast, the soils planted with soybeans and maize had the highest relative abundances of *Basidiomycota* (13.52%) and *Mortierellomycota* (13.71%), respectively (Figure 3b).

The Bray–Curtis NMDS analysis indicated that the crop planted influenced the composition of the soil microbial communities (Figure 3c, stress = 0.1383; Figure 3d, stress = 0.1930). The composition of the bacterial and fungal communities differed significantly among crop types, according to PERMANOVA analysis. Nevertheless, the explanatory variation in the composition of the bacterial and fungal communities explained by the crop type was 18.34% and 13.35%, respectively (Figure 3c, R^2^ = 0.1835, *p* = 0.001; Figure 3d, R^2^ = 0.1292, *p* = 0.001). Crop types had a greater impact on the composition of the fungal community than on the bacteria, with sorghum- and cotton-planting soil showing significant differences in fungal community composition (Figure 3c,d).

### 3.3. Co-Occurrence Network Structure of Microbial Communities

**Figure 4:**
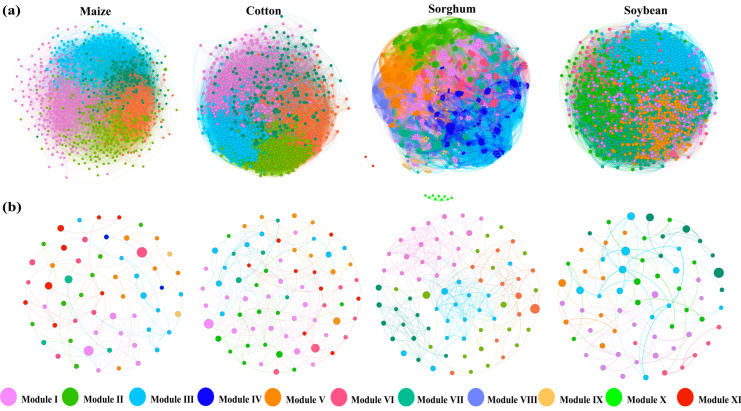
Co-occurrence networks of soil bacterial (**a**) and fungal (**b**) communities in different crop types. Nodes indicate operational taxonomic units (OTUs). Different modules are presented in various colors.

To elucidate the variations in interactions among soil bacterial populations under different crop types, OTUs were used to build co-occurrence networks of soil bacteria and fungi for each crop type (Figure 4a,b). Nodes and edges of the same color represent species and their connections within the same module. This study finds that the bacterial network was considerably more complex than the fungal network, while the sorghum network showcases even more intricate network modules. Based on the computation of the bacterial network’s topological features (Table 2), the soil bacterial co-occurrence network for sorghum- and soybean-planting soils comprised eight and seven modules, respectively. In contrast, maize- and cotton-planting soils each exhibited five modules. Comparing the sorghum-planting soil network with the other three groups, it demonstrated a more intricate fungal co-occurrence network, as evidenced by its higher average clustering coefficient (0.568), assortativity (0.643), transitivity (0.560), modularity (0.545), and average path length (3.091). The cotton-planting soil network, on the other hand, suggested a more stable bacterial co-occurrence network, characterized by the most complicated interactions, the highest number of significant positive and negative linkages, and the highest robustness score (0.171). The maize-planting soil bacterial network was distinguished by a more positive symbiotic relationship attributed to a higher proportion of positive links.

The topological characteristics of the fungal network reveal that the soil fungal co-occurrence network for planting maize, cotton, soybeans, and sorghum contains nine, seven, six, and five modules, respectively (Table 3). The sorghum-planting soil network displays a more intricate fungal co-occurrence network compared to the other three groups, as demonstrated by its higher average degree (8.513), clustering coefficient (0.519), assortativity (0.629), and net transitivity (0.610). A more stable fungal co-occurrence network was also found in sorghum-planting soil, as evidenced by the soil’s higher robustness score (0.128), more complicated interactions, and the greatest number of positive and negative linkages. In the soil fungal networks of soybean crops, the proportion of positive linkages was higher, indicating the existence of more beneficial symbiotic partnerships.

### 3.4. The Impact of Environmental Factors on Microbial Communities

**Figure 5:**
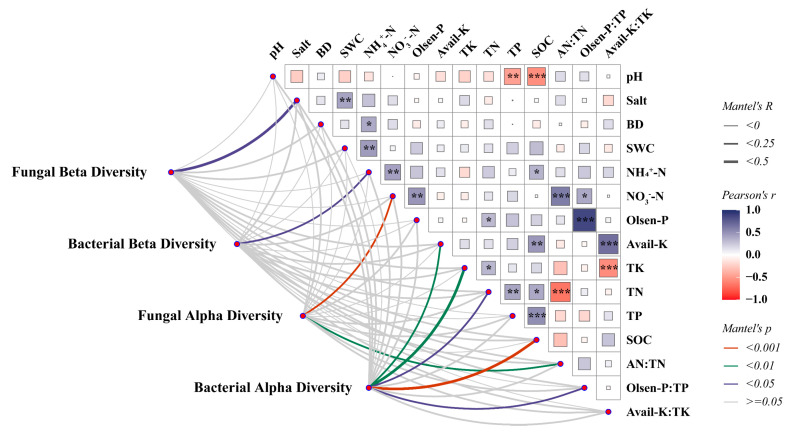
The Mantel test between environmental factors and microbial diversity. Line width is proportional to Mantel’s r statistic, and line color denotes statistical significance. Pairwise comparisons of environmental factors are also shown, with color gradient and square size denoting Pearson’s correlation coefficient. *, *p <* 0.05; **, *p <* 0.01; ***, *p* < 0.001.

The relationship between environmental factors and microbial diversity, as well as community composition, was explored by the Mantel test (Figure 5). We found that alpha diversity was more sensitive to environmental variation compared to beta diversity. Specifically, the fungal alpha diversity was mainly influenced by NO_3_^−^-N (r = 0.25, *p* = 0.001), while the bacterial alpha diversity was influenced by SOC (r = 0.26, *p* = 0.001), TN (r = 0.24, *p* = 0.011), TK (r = 0.32, *p* = 0.002), and Avail-K (r = 0.22, *p* = 0.01) content. However, there was a significant correlation between the alpha diversity of bacteria and Olsen-P: TP (r = 0.19, *p* = 0.029), indicating a relationship with soil phosphorus-use efficiency, and a significant correlation was found between the fungal alpha diversity and AN:TN (r = 0.21, *p* = 0.008), indicating a close relationship with soil nitrogen use efficiency. Additionally, soil salinity (r = 0.25, *p* = 0.039) affected the beta diversity of soil fungi, while ammoniacal nitrogen (NH_4_^+^-N) (r = 0.15, *p* = 0.037) influenced the beta diversity of soil bacteria.

### 3.5. The Impact of Microbial Communities on MNC

**Figure 6:**
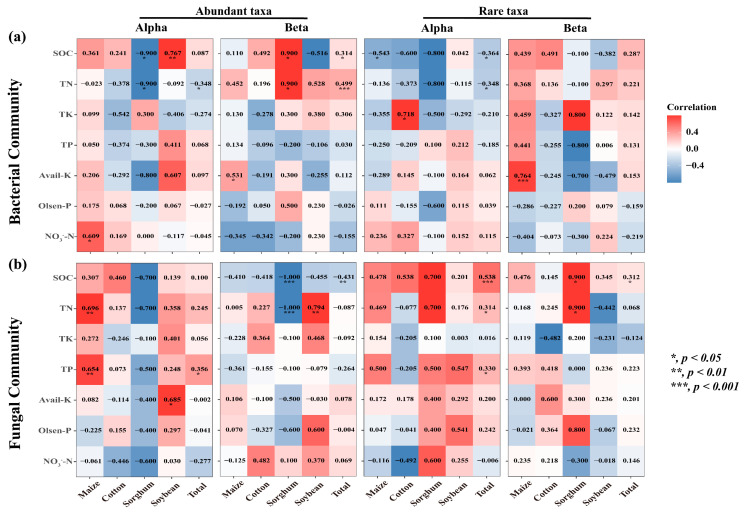
Heatmaps of correlation (Spearman’s) coefficients between the microbial alpha diversity and beta diversity of rare and abundant taxa and all individual nutrient variables. (**a**,**b**) denote the bacterial and fungal communities, respectively. The numbers in the table are r values. The shading from white to red represents a low-to-high positive correlation, while the shading from white to blue represents a low-to-high negative correlation. TN, total nitrogen; TK, total potassium; TP, total phosphorus; NO_3_^−^-N, nitrate-N; Olsen-P, available phosphorus; Avail-K, available potassium; SOC, soil organic carbon.

**Figure 7:**
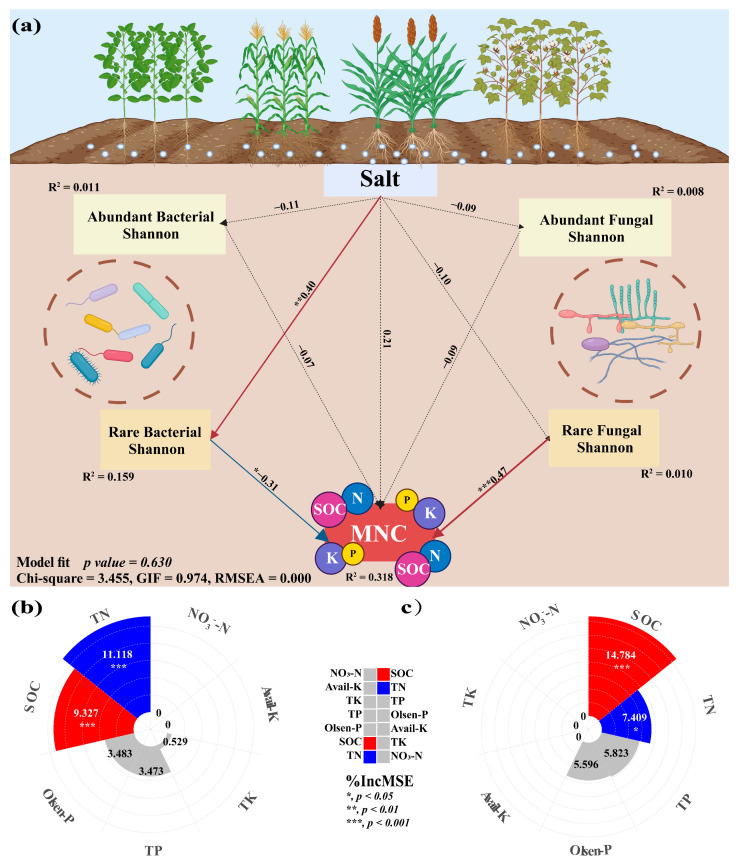
Structural equation model showing the causal relationship among soil salinity, the alpha diversity (Shannon index) of soil microbial rare and abundant sub-communities, and the multi-nutrient cycling index (*MNC*) (**a**). Random forest model reveals optimal predictors of the bacterial and fungal rare taxa alpha diversity (Shannon index) to the constituent indicators of soil multi-nutrient cycling (**b**,**c**) in the different crop-planting soil. Red solid lines indicate significant positive relationships; blue solid lines indicate significant negative relationships. Black dotted lines indicate insignificant relationships. *, *p* < 0.05; **, *p* < 0.01; ***, *p* < 0.001; TN, total nitrogen; TK, total potassium; TP, total phosphorus; available phosphorus; SOC, soil organic carbon.

The findings demonstrated the significant influence of crop types on the correlation between nutritional variables and the diversity of rare and abundant taxa in soil. The alpha and beta diversity of soil bacterial-abundant taxa from sorghum-planting significantly affected SOC (Alpha: r = −0.9, *p* = 0.037; Beta: r = 0.9, *p* = 0.037) and TN (Alpha: r = −0.9, *p* = 0.037; Beta: r = 0.9, *p* = 0.037) contents. Alpha was negatively correlated, and beta was positively correlated. The rare taxa beta diversity of sorghum soil fungi also significantly affected SOC (r = 0.9, *p* = 0.037) and TN (r = 0.9, *p* = 0.037) content, and both were positively correlated. Notably, there was a substantial correlation between rare taxa alpha diversity and the contents of SOC (Bacterial: r = −0.364, *p* = 0.019; Fungal: r = 0.538, *p* < 0.001) and TN (Bacterial: r = −0.348, *p* = 0.026; Fungal: r = 0.314, *p* = 0.045) (Figure 6). This study investigated the role of rare and abundant taxa alpha diversity in soil microbial diversity in the *MNC*. In the SEM analysis, we considered the alpha diversity of rare and abundant taxa, soil salt, and *MNC* (Figure 7a). The findings demonstrated that the alpha diversity of rare bacterial taxa was strongly impacted by soil salts (path coefficient = 0.40, *p* = 0.006). Both rare bacterial (path coefficient = −0.31, *p* = 0.036) and fungal (path coefficient = 0.47, *p* < 0.001) taxa’s alpha diversity had a significant impact on the *MNC*. It showed a negative correlation between *MNC* and a greater alpha diversity index of rare bacterial taxa. Conversely, the alpha diversity index for rare fungal taxa showed the reverse trend. The alpha diversity (Shannon index) of rare taxa has been predicted using the random forest model to account for the impact of soil multi-nutrient cycling compositional indicators (Figure 7b,c). According to the findings, the primary determinants of alpha diversity among soil rare taxa were SOC (Bacterial: 9.327%, Fungal: 14.784%) and TN (bacterial: 11.118%, fungal: 7.409%) content.

## 4. Discussion

### 4.1. Impact of Different Crop Types on Soil Microbial Diversity and Community Composition

Microbial community diversity and composition are determined mainly by the physicochemical and biological characteristics of the soil, such as soil type and texture, soil pH and moisture, soil management techniques, crop types, and seasonal and climatic circumstances [29,30,31]. Soil bacteria show more significant differences than fungal diversity. Because bacteria are more sensitive to environmental changes while fungi are more adaptable to long-term stable soil conditions. Furthermore, bacterial communities show more variety than fungi in complex cropping systems (such as continuous or rotational cropping) [32,33,34]. Different crop types have different root structures, secretions, and residues [35], affecting soil physicochemical properties and greatly influencing the structure and diversity of soil microbial communities [36]. For example, Neha et al. (2022) found differences in soil microbial biomass and microbial community structure across crops (chickpea, mustard, soybean, and maize) in tropical agroecosystems [35]. Aurelie et al. (2019) studied the effects of four crop species (viz. clover, black oat, phacelia, tillage radish) on soil structure and microbial communities, finding that root phenotypic variations influenced soil porosity, aggregate structure, and microbial profiles [37]. In this study, the differences in diversity were attributed to the soil physicochemical characteristics, planting intensity, and root traits among crop types. Cotton-planting soil exhibited high nutrient conversion efficiencies and significant advantages in terms of endemic species and alpha diversity. Sorghum-planting soils with high nutrient conditions had low microbial alpha diversity indices and low numbers of endemic species. The high nutrient conditions may have facilitated the expansion of specific dominant populations, causing them to dominate ecosystem resources and suppressing the growth of other taxa [38]. It may also be caused by differences in field management [35].

Finally, the findings support the first hypothesis that the composition of soil microbial community and diversity varies according to crop type. Additionally, a substantial correlation was found between soil phosphorus and nitrogen levels and the alpha diversity of soil bacteria and fungi. As previously reported, these results are consistent with their different roles in the nutrient cycle process [17,18]. Alpha diversity of soil bacteria and fungus in agroecosystems (North China Plain) may be related to the availability of various nutrients (such as nitrogen and phosphorus) in the soil. The varying roles and nutrient needs of bacteria and fungi within the environment are reflected in this connection [39,40,41].

### 4.2. Impact of Different Crop Types on the Topology Character of Soil Microbial Co-Occurrence Networks

Co-occurrence network can better understand the ecological niche space among soil microbial communities by gaining new insights into the relationships between species [42]. The bacterial networks exhibited greater complexity in their nodes and connections compared to fungal networks. However, fungal networks demonstrated larger diameters and module values. The intricate structure of bacterial networks suggests a higher level of diversity, interaction, and adaptability within soil and other ecological environments. Conversely, the larger diameters and module values of fungal networks indicate a relatively independent and stable functional partitioning within the ecosystem [43]. Additionally, the microbial network structures varied significantly across different crop types.

In our study, soil bacterial and fungal networks among various crop types showed different structural patterns. This may be explained by the fact that various crop types substantially impact how soil microbial co-occurrence networks are configured, which changed the soil’s physicochemical characteristics and biological habitats. This discrepancy indicated the microbial community’s capacity to adjust to shifting environmental conditions and their ecological functions [42,44,45]. Cotton-planting soil exhibited a complex bacterial network with high information transmission potential and robustness. These characteristics contribute to superior nutrient transformation efficiency, enhancing soil stability and adaptability while promoting efficient nutrient cycling. Sorghum-planting soil exhibited a bacterial network with high modularity and functional differentiation, while the fungal network displayed structural complexity and robustness, indicating effective microbial organization. The soil’s nutrient conditions were also relatively favorable with a high *MNC*, indicating efficient nutrient cycling. These factors suggest that the microbial network in sorghum soil contributes to effectively managing soil nutrients, supporting both nutrient availability and soil health. However, its low robustness, which the sorghum bacterial community might have depended on to sustain specific key microbial populations, made it less resistant to environmental disturbances. The stability of the soil’s microbial network is vital for an agroecosystem’s long-term health, as it directly impacts soil nutrient-use efficiency, ecological balance, and crop productivity [45]. Although it performed relatively poorly in robustness, its network complexity was the largest. This also implies an inverse relationship between the network complexity of soil microbial communities and their ability to maintain stability [46]. An analysis of wheat’s inter-root soil microbial networks in the eastern Qinghai–Tibetan Plateau and the North China Plain has revealed a similar pattern [47]. Microorganisms’ symbiotic connections have the potential to make co-occurring networks more complicated. Microbes may cooperate to endure adverse conditions by developing stronger, mutually advantageous bonds when confronted with environmental stressors. These connections may help create networks that are more adaptable and sophisticated [48]. Soil multi-nutrient cycle processes are intimately linked to complex symbiotic connections in soil microbial co-occurrence networks [49]. Consequently, one of the key elements influencing soil multi-nutrient cycling is the intricacy of the soil microbial co-occurrence network.

### 4.3. Impact of Different Crop Types on the Relationship Between Microbial Taxa Diversity and Soil Nutrients

Soil microorganisms exhibit significant differences in the abundance of both rare and abundant taxa and their functional roles. Rare taxa serve as genetic information banks and are vital components of ecological functions, such as nutrient cycling and responding to environmental disturbances [11,50,51]. Compared to abundant taxa, rare taxa had a higher alpha diversity, and the diversity was highly connected with soil nutrient variables, particularly the SOC and TN content which emphasized the importance of soil carbon and nitrogen. Cui et al. (2023) found that rare taxa possessed unique metabolic functions under environmental stress, which enhanced nitrogen cycling [52]. Similarly, Cao et al. (2023) demonstrated that the diversity of rare bacteria was closely linked to carbon decomposition, with functional redundancy significantly contributing to carbon cycling-related processes [18]. These findings underscore the critical role of rare microbial taxa in maintaining soil health and supporting nutrient cycling. The diversity of rare and abundant soil taxa in sorghum had stronger correlations with TN and SOC. The diversity of rare and abundant taxa varies across crop types regarding soil nutrient levels. Additionally, the differences in root structures and the unique chemical secretions associated with each crop type can influence the composition of soil microbial communities [53], leading to changes in both rare and abundant taxa. However, further research should also focus on optimizing soil microbial communities according to different crop types to enhance soil nutrients’ cycling efficiency, thus providing a theoretical basis for crop management practices and promoting sustainable agricultural development.

The second hypothesis, that the diversity of taxa, both rare and abundant, showed different responses to soil nutrients regarding various crop types, is thus supported by our study. We also discovered that the alpha diversity of rare taxa was more susceptible to soil nutrient changes. Therefore, it is crucial to concentrate on the role of abundant taxa in nutrient cycling and to give additional thought to the role of rare taxa when optimizing agricultural management practices to preserve the stability of microbial communities and soil functioning [54,55].

### 4.4. Impact of Abundant and Rare Microbial Taxa Diversity on Soil Multi-Nutrient Cycling Index

The result of SEM showed that the alpha diversity of rare taxa was more susceptible to variations than abundant taxa in soil salinity. The sensitivity of rare taxa to environmental change has been demonstrated in other ecosystems, such as salt marsh ecosystems [56]. On the other hand, in saline and agricultural systems, abundant taxa show more significant dispersal limitation than rare taxa [57]. This could be explained by the study’s sample plots’ soil salinity intervals, primarily concentrated in mild salinity. The diversity of microorganisms is closely related to the cycling of multiple nutrients in terrestrial ecosystems, particularly in alpha diversity [58,59]. In this study, the *MNC* was significantly affected by the alpha diversity of rare taxa. Conversely, *MNC* was not significantly influenced by the alpha diversity of abundant taxa. The hypothesis proposed may be attributed to the fact that rare taxa play a pivotal role in numerous other soil processes, including the nitrogen–phosphorus–carbon cycle and ecological functioning systems. Rare taxa have demonstrated their significance in ecological functioning systems [55]. Consequently, preserving the diversity of soil microbial communities, particularly that of rare taxa, is imperative to guarantee sustainable agroecosystems in the future.

Abundant taxa are important executors in soil multi-nutrient cycling, but our results indicated a significant relationship between rare taxa and *MNC*. Although abundant taxa contribute to the most of soil functions, the difference of nutrient cycling in different habitats was probably influenced by rare taxa. Rare microbial taxa demonstrate specialized metabolic capabilities to promote nutrient cycling under certain conditions or in unique habitats [60]. The considerable physiological and morphological plasticity of fungi can improve soil nutrient availability and promote carbon and nitrogen cycling within ecosystems [54,61] as well as the breakdown of soil organic matter [62]. Importantly, rare fungal taxa may exhibit greater specialization in the decomposition of organic matter and the mineralization of nutrients [63], processes that directly enhance *MNC*. In conclusion, the diversity of rare taxa significantly impacts *MNC*.

## 5. Conclusions

This study thoroughly assessed the alterations in the composition, diversity, and network structure of soil microbial communities, as well as the primary impacting factors, across four crop types: maize, soybean, sorghum, and cotton in the YRD. The diversity of rare and abundant taxa in soils across different crop types varies in response to soil nutrient factors. Differences in crop types and the interactions between microbial communities all influence the diversity of rare and abundant taxa. Moreover, carbon and nitrogen levels are key determinants in shaping the diversity of rare taxa, influencing overall microbial composition and the efficiency of nutrient cycling. The findings of this study suggested rare taxa as key drivers of soil multi-nutrient cycling under different crop types. Optimizing crop-planting patterns, adjusting the structure of soil microbial communities, and promoting the activity of rare groups can enhance land productivity and improve soil nutrient cycling efficiency in saline-alkali conditions. This approach can deepen our understanding of microbial communities functional and buffering capacities in cultivated saline-alkali environments.

## Figures and Tables

**Table 1:** Soil physicochemical properties under different crop types.

Soil Properties	Soybean	Sorghum	Cotton	Maize
pH	8.64 ± 0.08a	8.53 ± 0.02a	8.65 ± 0.07a	8.59 ± 0.05a
Salt (‰)	0.76 ± 0.13b	1.34 ± 0.19ab	1.69 ± 0.26a	0.74 ± 0.12b
BD (g cm^−3^)	1.37 ± 0.02a	1.46 ± 0.03a	1.43 ± 0.03a	1.39 ± 0.03a
SWC (%)	11.51 ± 1.17b	18.70 ± 1.73a	16.29 ± 1.25ab	14.07 ± 1.58ab
NH_4_^+^-N (mg kg^−1^)	1.29 ± 0.09b	2.75 ± 0.24a	2.53 ± 0.10a	2.56 ± 0.10a
NO_3_^−^-N (mg kg^−1^)	45.6 ± 13.87a	99.0 ± 9.98a	79.7 ± 13.97a	78.5 ± 9.87a
Olsen-P (mg kg^−1^)	14.90 ± 4.18a	22.54 ± 5.87a	28.13 ± 8.23a	17.13 ± 2.83a
Avail-K (mg kg^−1^)	116 ± 5.93a	135 ± 19.26a	158 ± 14.70a	149 ± 16.25a
AN (g kg^−1^)	0.05 ± 0.01a	0.10 ± 0.01a	0.08 ± 0.01a	0.08 ± 0.01a
TP (g kg^−1^)	2.75 ± 0.34a	2.96 ± 0.33a	2.51 ± 0.24a	3.13 ± 0.37a
TK (g kg^−1^)	18.2 ± 2.10b	24.3 ± 3.20a	14.7 ± 1.58b	15.6 ± 1.28b
TN (g kg^−1^)	1.46 ± 0.43b	6.23 ± 2.20a	1.64 ± 0.62b	2.14 ± 0.46b
SOC (g kg^−1^)	5.76 ± 0.36a	8.69 ± 1.22a	6.87 ± 0.73a	7.66 ± 0.72a
AN: TN (%)	4.54 ± 1.34a	3.81 ± 1.63a	8.41 ± 1.66a	8.17 ± 2.13a
Olsen-P: TP (%)	0.54 ± 0.15a	0.83 ± 0.25a	1.01 ± 0.23a	0.68 ± 0.16a
Avail-K: TK (%)	0.69 ± 0.06b	0.62 ± 0.15b	1.19 ± 0.15a	0.96 ± 0.07ab
MNC	0.25 ± 0.03b	0.48 ± 0.07a	0.33 ± 0.03b	0.35 ± 0.04b

Mean values (means ± SD) followed by different letters indicate a significant difference between crop types at *p* < 0.05 (*p* < 0.05; multiple comparison by Duncan’s test).

**Table 2:** Topological properties of the co-occurrence networks of the bacterial community in soils of different groups.

Network Parameters	Soybean	Sorghum	Cotton	Maize
Average Degree	86.492	83.845	116.384	54.567
Average Weighted Degree	62.562	78.088	82.349	37.3
Diameter	3	5	4	4
Average Path length	2.206	3.091	2.074	2.426
Density	0.071	0.067	0.114	0.056
Modularity	0.358	0.545	0.309	0.391
Number of Communities	7	8	5	5
Average Clustering Coefficient	0.351	0.568	0.422	0.345
Node_num	1211	1254	1021	970
Edge_num	52,371	52,571	59,414	26,465
Net. transitivity	0.363	0.56	0.472	0.383
Assortativity	0.448	0.643	0.493	0.485
Net. degree.centrality	0.077	0.076	0.176	0.128
Average_neighbors	92.993	87.035	133.578	65.166
Net. positive	0.646	0.524	0.588	0.825
Net. negative	0.354	0.476	0.412	0.175
Robustness	0.083	0.086	0.171	0.092

**Table 3:** Topological properties of the co-occurrence networks of the fungal community in soils of different groups.

Network Parameters	Soybean	Sorghum	Cotton	Maize
Average Degree	4.493	8.513	5.333	3.069
Average Weighted Degree	3.206	7.902	3.676	2.12
Diameter	8	11	7	10
Average Path length	3.514	4.052	3.094	4.431
Density	0.066	0.111	0.072	0.054
Modularity	0.609	0.545	0.52	0.652
Number of Communities	6	5	7	9
Average Clustering Coefficient	0.363	0.519	0.345	0.278
Node_num	69	78	75	58
Edge_num	155	332	200	89
Net. transitivity	0.288	0.608	0.317	0.431
Assortativity	0.0572	0.629	0.148	0.553
Net. degree.centrality	0.066	0.11	0.104	0.087
Average_neighbors	5.3	9.428	6.435	3.641
Net. positive	0.697	0.569	0.665	0.652
Net. negative	0.303	0.431	0.335	0.348
Robustness	0.038	0.128	0.044	0.044

## Data Availability

The datasets generated during the current study are available in the https://figshare.com/articles/dataset/The_original_data_and_images_of_the_article_Rare_rather_than_abundant_microbial_taxa_diversity_drives_soil_multi-nutrient_cycling_under_different_crop_types_/27710133 (accessed on 20 June 2023).

## Supplementary Materials

The following supporting information can be downloaded at: https://www.mdpi.com/article/10.3390/microorganisms13030513/s1. Included; The methods of soil physicochemical properties; High-throughput Sequencing and bioinformatics analysis; Table S1: Soil network cohesion values under different crop types; Table S2: Shannon index of soil abundant and rare taxa. Refs. [64,65,66] can be found in Supplementary Materials.
