# Supplementary material for: Rare Taxa as Key Drivers of Soil Multi-Nutrient Cycling Under Different Crop Types
Source: Microorganisms. 2025 Feb 26;13(3):513. doi: 10.3390/microorganisms13030513 (PMC11944799; doi:10.3390/microorganisms13030513)
Supplement: Supplementary file 1 [file microorganisms-13-00513-s001.zip › microorganisms-3402211-supplementary.pdf]

Rare taxa as key drivers of soil multi-nutrient cycling under different crop types

Qingmiao Yang [1,2,#], Hanwen Liu [2,#], Biao Tang [3], Chunxiao Yu [2], Shide Dong [2], Yang Li [2], Guangxu Cui [2], Yi Zhang [2], Guangmei Wang [2*]

1 School of Life Sciences, Ludong University, Yantai 264025, China

2 CAS Key Laboratory of Coastal Environmental Processes and Ecological Remediation, Yantai Institute of Coastal Zone Research, Chinese Academy of Sciences, Yantai 264003, China.

3 CCCC-FHEC Ecological Engineering Co.， Ltd， Shenzhen 518106, China.

* Corresponding authors: Prof. Guangmei Wang

Yantai Institute of Coastal Zone Research, Chinese Academy of Sciences, Yantai 264003, China

Tel.: +86- 05352109118

E-mail address: gmwang@yic.ac.cn (Guangmei Wang)

[#]Qingmiao Yang and Hanwen Liu contributed equally to this work.

The methods of soil physicochemical properties

Soil electrical conductivity (EC) and pH were determined in a 1:5 soil/water suspension using a pH meter and an EC meter, respectively. Soil bulk density (BD) was measured at 0–20 cm soil depths of each quadrat using a cutting ring with 100 cm^{-3} volume [1]. The content of NO_3^--N and NH_4^+-N were extracted by 2 mol/L KCl solution, and measured by the continuous flow analytical system at wavelengths 660 nm and 520 nm (AA3, Germany)[2]. Olsen-phosphorus (Olsen-P) was extracted by 0.5 mol/L Na_2CO_3 and determined by molybdenum blue method. The content of available potassium (Avail-K) was extracted with 1 mol/L NH_4Ac and estimated by an atomic absorption spectrophotometer (TAS-990, China). Soil organic carbon (SOC) was analyzed using the dichromate oxidation method. After digestion with H_2SO_4, soil total nitrogen (TN) and total phosphorus (TP) were measured using the continuous flow analyzer at wavelength 660 nm (AA3, Germany). Total potassium (TK) was estimated using an atomic absorption spectrophotometer (TAS-990, China).

Table S1. Soil network cohesion values under different crop types.

	cohesion	Soybean	Sorghum	Cotton	Maize
**Bacterial network**	cohesion.pos	0.724(0)	0.931(0)	0.706(0.001)	0.68(0.001)
	cohesion.neg	-0.715(0)	-0.93(0)	-0.698(0.001)	-0.643(0.002)
**Fungal network**	cohesion.pos	0.69(0.005)	0.854(0.022)	0.672(0.004)	0.603(0.013)
	cohesion.neg	-0.382(0.04)	-0.838(0.032)	-0.625(0.019)	-0.39(0.033)

Table S2. Shannon index of soil abundant and rare taxa.

Shannon index	Rare taxa	Abundant taxa
Bacteria	6.51(0.04)	5.09(0.04)
Fungus	4.24(0.07)	3.33(0.06)

High-throughput Sequencing and bioinformatics analysis

Bacterial 16SrRNA genes were amplified using primer pairs 515F (5'-GTGCCAGCMGCCGCGG-3') with 907R (5'-CCGTCAATTCMTTTRAGTTT-3'), respectively, and the ITS1 fungal region was amplified using primer pairs ITS86F (5'-GTGAATCATCGAATCTTTGAA-3') with ITS4R (5'- TCCTCCGCTTATTGATATGC-3').

We obtained the high-quality sequenced data of bacteria (N¼ 548,708) and fungi (N¼ 614,171) after the removal of short fragments (<200bp) and low-quality sequences (average quality scores <25 reads) by using Quantitative Insight into Microbial Ecology (QIIME-1.9.1) pipeline (http://qiime.sourceforge. net/) [3]. OTUs were generated based on a 97% level of similarity using UCLUS [4]. We used the SILVA database (https://www.arb-silva.de/) for the taxonomic identification of each phylotype of bacteria, and we used the UNITE database (https://unite.ut.ee/) for the taxonomic identification of fungi.

References

1. Kong, B.; Zhu, T.; Ming, Y.; Jia, S.; Li, C.; Wang, F.; Dong, Z.; Jiao, S.; Li, Y.; Shi, L. Effects of Three Long-Term Land Use Patterns on Soil Degradation in the Yellow River Delta: Evidence from Ecological Stoichiometry. *Agronomy* **2023**, *13*, 2744, doi:10.3390/agronomy13112744.

2. Jones, D.L.; Willett, V.B. Experimental Evaluation of Methods to Quantify Dissolved Organic Nitrogen (DON) and Dissolved Organic Carbon (DOC) in Soil. **2005**, doi:10.1016/j.soilbio.2005.08.012.
3. Caporaso, J.G.; Kuczynski, J.; Stombaugh, J.; Bittinger, K.; Bushman, F.D.; Costello, E.K.; Fierer, N.; Peña, A.G.; Goodrich, J.K.; Gordon, J.I.; et al. QIIME Allows Analysis of High-Throughput Community Sequencing Data. *Nat Methods* **2010**, *7*, 335–336, doi:10.1038/nmeth.f.303.
4. Edgar, R.C. Search and Clustering Orders of Magnitude Faster than BLAST. *Bioinformatics* **2010**, *26*, 2460–2461, doi:10.1093/bioinformatics/btq461.
